# Towards Raman-Based Screening of Acute Lymphoblastic Leukemia-Type B (B-ALL) Subtypes

**DOI:** 10.3390/cancers13215483

**Published:** 2021-10-31

**Authors:** Patrycja Leszczenko, Aleksandra Borek-Dorosz, Anna Maria Nowakowska, Adriana Adamczyk, Sviatlana Kashyrskaya, Justyna Jakubowska, Marta Ząbczyńska, Agata Pastorczak, Kinga Ostrowska, Malgorzata Baranska, Katarzyna Maria Marzec, Katarzyna Majzner

**Affiliations:** 1 Faculty of Chemistry, Jagiellonian University, Gronostajowa 2, 30-387 Krakow, Poland; patrycja.leszczenko@doctoral.uj.edu.pl (P.L.); aleksandra.dorosz@doctoral.uj.edu.pl (A.B.-D.); anna.maria.nowakowska@uj.edu.pl (A.M.N.); adriana.adamczyk@doctoral.uj.edu.pl (A.A.); sviatlana.kashyrskaya@student.uj.edu.pl (S.K.); m.baranska@uj.edu.pl (M.B.); 2 Jagiellonian Centre for Experimental Therapeutics (JCET), Jagiellonian University, Bobrzynskiego 14, 30-348 Krakow, Poland; 3 Department of Pediatrics, Oncology and Hematology, Medical University of Lodz, Sporna 36/50, 91-738 Lodz, Poland; justyna.magdalena.jakubowska@umed.lodz.pl (J.J.); marta.zabczynska@umed.lodz.pl (M.Z.); agata.pastorczak@umed.lodz.pl (A.P.); kinga.ostrowska@umed.lodz.pl (K.O.); 4 Lukasiewicz Research Network—Krakow Institute of Technology, Zakopiańska 73, 30-418 Krakow, Poland

**Keywords:** Raman spectroscopy, acute lymphoblastic leukemia (ALL), fusion genes, chemometric techniques, *BCR-ABL1*, *TCF-PBX1*, *TEL-AML1*

## Abstract

**Simple Summary:**

Acute lymphoblastic leukemia (ALL) is the most common pediatric malignancy originating from abnormal lymphoid progenitor cells. Since ALL is genetically highly heterogenous, more sensitive and rapid methods for identifying the molecular subtype of ALL are still being searched, and Raman spectroscopy (RS) has a chance of becoming a valuable tool for this purpose. Herein, the RS was applied to analyze normal B cells and three subtypes of B-ALL, characterized by the presence of the product of gene fusion, i.e., *BCR-ABL1, TEL-AML1*, and *TCF3-PBX1*. The classification and discrimination of normal and neoplastic cells were carried out with the chemometric approach. Normal B cells were characterized mostly by bands assigned to nucleic acids and proteins, whereas three subtypes of ALL appeared to contain a higher lipid content. Spectral differences between particular ALL subtypes were modest. The results lead to the conclusion that RS has the potential as a diagnostic tool in clinical practice.

**Abstract:**

Acute lymphoblastic leukemia (ALL) is the most common type of malignant neoplasms in the pediatric population. B-cell precursor ALLs (BCP-ALLs) are derived from the progenitors of B lymphocytes. Traditionally, risk factors stratifying therapy in ALL patients included age at diagnosis, initial leukocytosis, and the response to chemotherapy. Currently, treatment intensity is modified according to the presence of specific gene alterations in the leukemic genome. Raman imaging is a promising diagnostic tool, which enables the molecular characterization of cells and differentiation of subtypes of leukemia in clinical samples. This study aimed to characterize and distinguish cells isolated from the bone marrow of patients suffering from three subtypes of BCP-ALL, defined by gene rearrangements, i.e., *BCR-ABL1* *(Philadelphia*-positive, t(9;22)), *TEL-AML1* (t(12;21)) and *TCF3-PBX1* (t(1;19)), using single-cell Raman imaging combined with multivariate statistical analysis. Spectra collected from clinical samples were compared with single-cell spectra of B-cells collected from healthy donors, constituting the control group. We demonstrated that Raman spectra of normal B cells strongly differ from spectra of their malignant counterparts, especially in the intensity of bands, which can be assigned to nucleic acids. We also showed that the identification of leukemia subtypes could be automated with the use of chemometric methods. Results prove the clinical suitability of Raman imaging for the identification of spectroscopic markers characterizing leukemia cells.

## 1. Introduction

Acute lymphoblastic leukemia (ALL) is a hematological malignancy originating from abnormal lymphoid progenitor cells that accumulate in the bone marrow and invade peripheral blood and extramedullary sites. B-cell precursor ALL (BCP-ALL) accounts for 80–85% of ALL. It is classified into several molecular subtypes depending on the initiating genetic lesion and specific secondary aberrations, which lead to a unique gene expression profile and chemosensitivity of leukemic cells [1]. Therefore, cytogenetic and molecular identification of the prognostically relevant genetic abnormalities became a routine diagnostic procedure that determines patients’ risk stratification and, consequently, an intensity of ALL treatment [2]. *TEL-AML1* translocation [t(12;21) (q13;q22)], *TCF3–PBX1* translocation [t(1;19)(q23;p13)], *KMT2A (MLL)* rearrangements (*KMT2A*-R), *BCR–ABL1* [*Philadelphia* chromosome (Ph) t(9;22)(q34;q11)-positive] are the most recurrent disease-initiating genetic alterations in BCP-ALL. *TEL-AML1* accounts for 25% of childhood cases and is related to an excellent prognosis. Similarly, *TCF3–PBX1* positive ALL also shows a favorable outcome but increases the risk for ALL relapse in the central nervous system. In contrast, *Ph*-positive ALL, which occurs ten times more rarely in children as compared to adults, who represent 40–50%, is clearly associated with poor prognosis [3]. The unfavorable outcome of this subtype results from the constitutive activity of the *BCR-ABL1* oncoprotein. This aberrant tyrosine kinase transfers a phosphate group to target molecules. It modifies a phosphorylation pattern in numerous signaling pathways in response to external stimuli, strongly enhancing the proliferation of leukemic cells.

Since genetic subtypes of BCP-ALL display distinct gene expression profiles, reflecting activation of particular metabolic pathways, leukemic cells may be easily distinguished from normal B cells and each other using high-throughput genomic methods. Moreover, ALL cells can be sorted out by flow cytometry based on the expression of surface proteins (immunophenotype). However, using both methods, pathological lymphoblasts are subjected to invasive procedures of sample preparation which either destroy cells or significantly disturb their homeostasis, making it impossible to reuse the sample for further research. This study aims to investigate whether primary BCP-ALL cells representing defined genetic subgroups can also be discriminated based on their chemical composition using non-invasive and label-free Raman imaging. This technique measures molecular bond vibrations based on the two-photon scattering phenomenon resulting from the interaction of electromagnetic radiation with a sample, the annihilation of one photon, and the simultaneous creation of a second photon. The difference in energy between the incident photon and the Raman scattered photon is defined as the Raman shift (expressed as cm^−1^) [4]. Analysis of the intensity profile of the inelastically scattered light as a function of frequency provides the unique spectroscopic fingerprint of a tissue sample which reflects detailed chemical composition with respect to the presence of nucleic acids, proteins, lipids, and carbohydrates [4]. As a result of Raman imaging, complex datasets containing large amounts of information are produced. Thus, appropriate chemometric methods must be implemented in the extraction and correct interpretation of this information. Such statistical methods, including principal component analysis (PCA), and *k*-means cluster analysis (KMCA), reduce the dimensionality in the dataset, allow to capture of very subtle chemical changes in analyzed samples and group spectra based on their similarities [4,5,6]. KMCA provides the possibility of grouping the spectra into clusters based on spectral similarity and identifying a common biochemical pattern of the studied samples [4]. PCA helps to identify data patterns by highlighting differences in groups of spectra while reducing data redundancy simultaneously [7]. In the PCA method, one of the first steps of the analysis requires calculating principal components (PCs), which dissolve the variances within the spectra data set [7]. Recently, partial least squares (PLS) regression has been widely applied as a supervised chemometric technique for Raman data classification. It combines features from principal component analysis and multivariate regression and allows to build of predictive multicollinear models [8,9]. PLS regression is a powerful chemometric method, which serves as a supervised technique for generating machine learning prediction algorithms, which learn similarities and differences between classes of chemical data. In this method, known data (training set) are used to build a model, which in turn can be used to predict new, unknown data (validation samples) [8,9].

There are many advantages of Raman spectroscopy that justify its successful implementation into cancer cell profiling for routine clinical pathology practice and live-cell imaging. In addition to the high sensitivity and chemical specificity, using minimal or no sample pretreatment, small sample volume, and the possibility to perform measurements in liquids, Raman imaging allows nondestructive, intrinsic, and label-free cell characterization with excellent spatial resolution [4,10]. This great potential of RS for non-invasive cancer diagnostics has been effectively tested in a wide range of solid tumors, including breast, skin, brain, lung cancers, and others [11,12,13,14]. Raman studies on cancer detection have been predominantly focused on analyzing tissue sections, and only a limited number of experimental works were conducted on blood malignancies. The Raman spectroscopy-based approach of pathological cells analysis was previously reported, including a mixture with normal hematopoietic cells suspended in the solution [15,16] or B-ALL cell lines compared with normal B cells [17,18,19]. In the present work, we have reported for the first time that Raman spectroscopy was not only able to differentiate leukemic cells from normal lymphocytes, but it also enabled accurate classification of B-cell acute leukemia into the different differentiation/maturation stages [20]. It was also shown that the spectra of B-cell leukemia cell lines are characterized by a lower ratio of the intensity of the DNA/protein bands in relation to the healthy cells [18]. Thus, in the current study, we attempt to further characterize ALL cells being at the same stage of maturation (pre-B), depending on the presence of the specific genetic abnormalities, including *BCR-ABL1, TEL-AML1, TCF3-PBX1* gene fusions. The clinical usefulness of Raman imaging and spectroscopic markers for the characterization of leukemic cells compared to normal B cells is also shown.

## 2. Materials and Methods

### 2.1. B Lymphocytes and Cancer Cells Isolation

The experiments were performed in accordance with the consent of the Bioethics Committee at the Medical University of Lodz No. RNN/270/19/KE (extension KE/30/21) from 14 May 2019. Blood from volunteers and the bone marrow from the patients were collected after obtaining informed consent.

**Figure 1:**
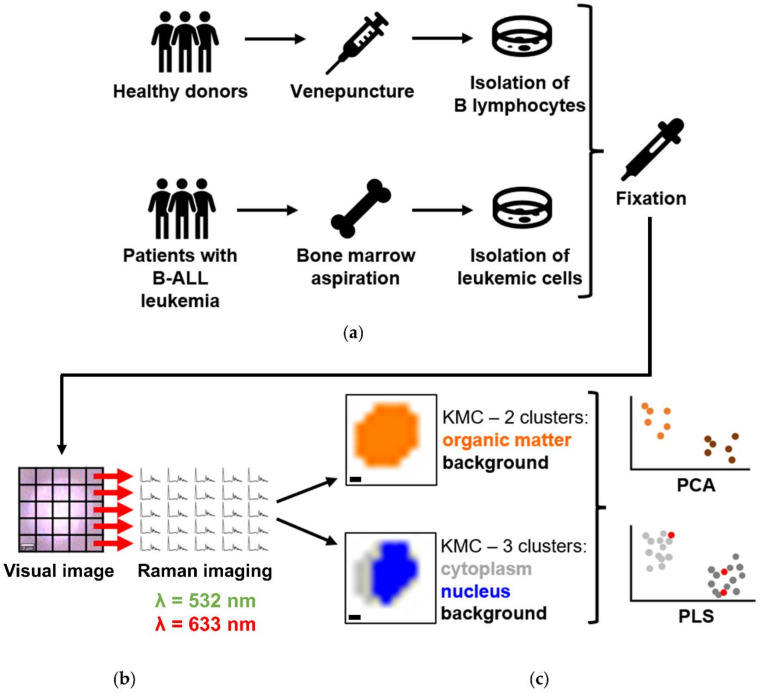
Scheme of conducted experiments. (**a**) Blood samples were drawn from healthy donors via venipuncture, and B cells were isolated. Leukemic cells were isolated from the bone marrow aspirate on the day of diagnosis of BCP-ALL and deposited in the Bio-Bank from patients with B-ALL. All cells were fixed with 0.5% GA and became a subject of (**b**) Raman imaging with two laser excitation wavelengths: 532 nm and 633 nm. Scale bar: 1 μm. (**c**) Subsequently, the acquired spectra were grouped using KMCA considering two approaches: deriving two clusters—organic matter and background, and 3 clusters—cytoplasm, nucleus, and background. After that, three groups of spectra were analyzed using PCA and PLS.

B lymphocytes were isolated from the whole peripheral blood of healthy donors (*n* = 5) (Figure 1a) with the immunomagnetic negative selection method (EasySep™ Direct Human B Cell Isolation Kit, STEMCELL Technologies Inc., Vancouver, CA, USA), enabling isolation of untouched target cells. The trypan blue exclusion method was used to assess the viability of isolated cells. The purity of obtained cells was evaluated with flow cytometry following staining with antibody cocktail: CD45-PerCP-Cy5.5 (clone 2D1) and CD19-APC (clone SJ25C1) (Figure S1). Primary leukemic cells were isolated from the bone marrow on the day of diagnosis of childhood BCP-ALL and were stored at −156 °C in the vapor phase of the liquid nitrogen. The Ficoll-Paque method was used for PBMC isolation according to the standard protocol. For this study, we selected only samples with three gene rearrangements typical for BCP-ALL, i.e., *BCR-ABL1*, *TEL-AML1*, *TCF3-PBX1*. Before Raman measurements, B-ALL samples were removed from the vapor phase of the liquid nitrogen and thawed quickly in a water bath (37 °C). After washing step with PBS (three times) at room temperature (300 g, 5 min), the viability of leukemic samples was assessed using the trypan blue exclusion method. The characteristics of selected samples is presented in Table S1. Both B lymphocytes and cancer cells were fixed with 0.5% glutaraldehyde (GA) for 10 min at room temperature and then washed three times with the use of PBS buffer to remove the excess amount of fixative. After that, cells were resuspended in the saline buffer for Raman measurements and kept at 4 °C.

### 2.2. Confocal Raman Imaging

Raman imaging of single cells was performed with the use of a confocal Raman microscope WITec Alpha 300 (WITec GmbH, Ulm, Germany) (Figure 1b) equipped with air-cooled 532 nm and 633 nm lasers, a CCD detector (Andor Technology Ltd., Belfast, Northern Ireland), which was cooled down to −60 °C and a 600 grooves/mm grating (BLZ = 500 nm) with a spectral resolution of around 3 cm^−1^. Cells (200–500 µL of cells suspensions), deposited on CaF_2_ windows (Crystran LTD, Poole, UK, Raman grade) and immersed in saline buffer were measured through illumination with the use of 63x water immersion objective (Zeiss W Plan-Apochromat 63x, NA = 1, Oberkochen, Germany). A 0.5 s exposure time per spectrum was applied for a green laser, and the sampling density was equal to 1 µm. For a red laser, spectra were collected with a step of 3 µm and the integration time was equal to 3 s. For statistical analysis, at least 50 cells/sample were measured from different areas of the sample. The cells were placed on CaF_2_ slides and measured at least after 30 min in order to let cells sediment and immobilize. Only normal-looking oval-shaped cells were measured.

### 2.3. Spectral Data Post-Processing and Analysis

At first, the spectra were pre-processed with Project Five 5.1 Plus software (WITec GmbH, Ulm, Germany). The pre-processing included the removal of artifacts from cosmic rays (cosmical ray removal, filter size: 3, and dynamic factor: 8) and background correction (polynomial fitting, 3rd order for a green laser, and 2nd order for a red laser). Then KMCA was performed, and two approaches were applied. Firstly, the averaged spectra of the whole-cell class were extracted. Secondly, spectra from each map of single cells were divided into two/three clusters characteristic for different cellular components, the class of nucleus, cytoplasm, lipid droplets, carotenoids, and hemoproteins (Figure 1c). Spectra were obtained from KMCA for each B-ALL subtype, and each class separately was averaged and presented with its standard deviation.

The chemometric analysis was performed with Unscrambler X v. software (v. 10.3, 64-bit, CAMO Software AS., Trondheim, Norway). Before applying the multivariate PCA, the spectra were smoothed (Savitzky–Golay, 3rd order polynomial, 15 pts both for green and red lasers), and baseline correction (linear correction and offset subtraction) and spectra normalization (unit vector normalization) were done. The PCA was performed in three different spectral ranges: fingerprint (1800–500 cm^−1^), C-H stretching region (3030–2850 cm^−1^), and in a wide range (3030–2850 cm^−1^ and 1800–500 cm^−1^). The PLS was executed in the spectral range of 1800–500 cm^−1^. In total, approximately 115,200 and 38,400 single spectra were analyzed using 532 nm and 633 nm lasers, respectively. It gives the overall number of single spectra of ca. 153,600.

## 3. Results

### 3.1. Characterization of the Molecular Composition of Normal B Lymphocytes and B-ALL Cells

Firstly, we performed Raman imaging of normal B-cells and their leukemic counterparts using 532 and 633 nm laser excitations. For each subtype among *BCR-ABL1*, *TEL-AML1*, *TCF3-PBX1*, 3–4 samples were collected from different patients (P1-10, Table S1).

**Figure 2:**
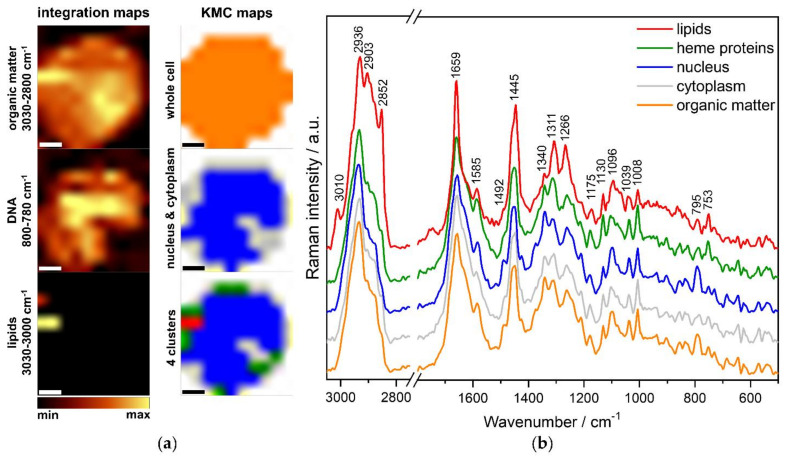
(**a**) Raman maps obtained using 532 nm laser constructed based on bands characteristic for organic matter (3030–2800 cm^−1^), nucleus (800–780 cm^−1^), and lipid droplets (3030–3000 cm^−1^) and KMCA. Scale bar: 2 μm. (**b**) Mean spectra for the classes differentiated from the KMC analysis for cells from an ALL patient with the *TCF3-PBX1* fusion gene. Spectra were maximally extended on the y axis and shown in the ranges 3050–2750 cm^−1^ and 1800–500 cm^−1^. Abbreviation: a.u.—arbitrary units.

Figure 2 presents the results of representative KMCA performed for Raman images of *TCF3-PBX1* positive blasts.

Figure 2a presents a set of Raman maps of exemplary lymphoblast. They were constructed based on the bands attributed to organic matter (3030–2800 cm^−1^), DNA/RNA (800–780 cm^−1^), and lipids (3030–3000 cm^−1^). The spatial distribution of organic matter allows visualizing the entire cell body and assessing cell morphology. Integration of bands characteristic for DNA/RNA is used to visualize the nucleus’s location and size. Analysis of the size and the distribution of lipid droplets (LDs) can give insight into the activity of cells because they are intracellular lipid reservoirs (fatty acids, triacylglycerides, and sterols), providing building components for membranes or substrates for energy metabolism [21,22]. Additionally, corresponding images were created as a result of KMC analysis—an organic matter of the whole cell (orange), two clusters representing spectra of nucleus and cytoplasm (blue and grey, respectively), and four clusters with other two groups derived from cytoplasm—lipid droplets (red) and hemoproteins (green).

Figure 2b displays the mean spectra for each cluster, representing distinct cellular components that differ from each other and exhibit characteristic bands. Identifying these clusters enables the characterization of the molecular structure of both normal and leukemic cells and the determination of their metabolic differences. Lipids are distinguished by bands characteristic for C-H stretching vibrations at 2936, 2903, and 2852 cm^−1^, and by the marker band at 3010 cm^−1^ originated from C=C groups, related to the degree of lipid unsaturation (Table 1). Additionally, the band at 1659 cm^−1^ is related to unsaturated lipids. Other bands used for lipids and proteins characterization are assigned to the CH_2_/CH_3_ in-plane bending at 1445 cm^−1^ and the CH_2_/CH_3_ wagging, twisting and/or bending at 1340 cm^−1^. Other characteristic bands of proteins at 1659, 1266, and 1039, 1008 cm^−1^, can be assigned to C=O stretching of α-helix (amide I), C-N stretching of α-helix (amide III), stretching of C-N groups, and ring breathing of phenylalanine, respectively. Amide I and III regions of RS spectra appear to be a valuable tool in estimating the secondary structure of proteins. Bands characteristic for hemoproteins (green) were observed at 1585, 1311, 1130, and 753 cm^−1^ and originated from C=C bending in phenylalanine, pyrrole asymmetric stretching, stretching of C-N groups, and breathing mode of the pyrrole ring, respectively. The unique band for the nucleus (blue) was 795 cm^−1^, which could be assigned to ring breathing of nitrogenous bases in nucleic acids (mainly cytosine). Another band, which could be assigned to nucleic acids, was the band at 1096 cm^−1^ (symmetric stretching of PO_2_^−^ groups of the DNA backbone). All the assignments and classifications of Raman bands are collected in Table 1.

Based on KMC analysis, the percentage of cells containing hemoproteins, carotenoids, and lipids was calculated in relation to each group’s total number of examined cells. The summary of Raman measurements of analyzed cells is presented in Table 2. Spectra of at least 50 cells per sample from each donor, using two lasers with the excitation of 532 nm and 633 nm, were gathered. The values representing the most abundant cluster within the analyzed cells are in bold.

Using a 532 nm laser excitation, in over 50% of the cells of each population, the resonance Raman profile of hemoprotein was detected [28,29]. The 56.6% of B cells and the 67.8% of *BCR-ABL1* leukemic cells were defined to include hemoproteins clusters. In the case of 633 nm laser excitation, the percentage of hemoprotein clusters was found at the level of 0.0–1.5%, which is related to much lower resonance enhancement of hemoproteins at this excitation [29]. Carotenoids are also resonantly enhanced when excited in the visible range. Still, due to their greater absorption when excited with 532 nm laser and sensitivity, their relatively more intense bands are observed when the measurement is carried out with a 633 nm laser excitation. In line with previous observations, no carotenoid clusters were found in B cells [35]. In contrast, carotenoids were found in all studied types of leukemic cells, at the level of 8.3% for *BCR-ABL1* up to 12.5% for *TEL-AML1* cells. Lipids were identified in all groups of examined cells, at the level of 1.2% for B cells and up to 21.0% for *TEL-AML1*. Blasts with *TEL-AML1* fusion show the highest content of both carotenoids (12.5%) and lipids (21.0%) content. Some discrepancies in amounts of specific compounds calculated from data collected using two different lasers excitations originated either from different levels of resonance enhancement or from different sampling densities, i.e., 1 μm for 532 nm and 3 μm for 633 nm lasers.

**Figure 3:**
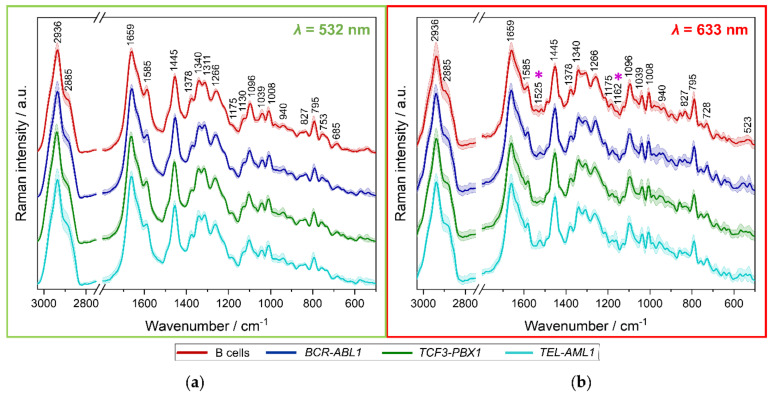
Mean spectra of the whole cells with the standard deviation, measured using a 532 nm laser (**a**) of B cells (*n* = 254), and BCR-ABL1 (*n* = 183), TCF3-PBX1 (*n* = 331), TEL-AML1 (*n* = 314) lymphoblasts; and using a 633 nm laser (**b**) of B cells (*n* = 247), and BCR-ABL1 (*n* = 169), TCF3-PBX1 (*n* = 271), TEL-AML1 (*n* = 254) lymphoblasts. Spectra were maximally extended on the y axis and shown in the ranges 3050–2750 cm^−1^ and 1800–500 cm^−1^. B cells are represented in red color, whereas leukemic cells with BCR-ABL1 fusion gene are in navy blue, with TCF3-PBX1 mutation are in green, and with TEL-AML1 fusion are in turquoise. In panel (**b**), bands at 1525 and 1162 cm^−1^ are marked with purple asterisks (*).

The next step of the analysis was a comparison of the average Raman spectra of normal B-cells and each subtype of leukemic cells. Figure 3 presents the average Raman spectra with the standard deviation obtained from whole cells of all four studied groups for 532 and 633 nm excitations.

Using only the visual assessment, the variation of the spectra presented in Figure 3 is imperceptible. The only noticeable differences can be noticed between the spectra of *BCR-ABL1* and *TEL-AML1* cells measured with a 633 nm excitation (Figure 3b, marked with purple asterisks). The difference is related to bands at 1525 and 1162 cm^−1^, corresponding to stretching of C=C and C-C groups of carotenoids, respectively (Table 1). To visualize and emphasize more subtle chemical changes in the registered spectra, we combined the RS analysis with PCA’s multivariate statistical method [21].

### 3.2. Spectroscopic Analysis of Different B-ALL Subtypes

**Figure 4:**
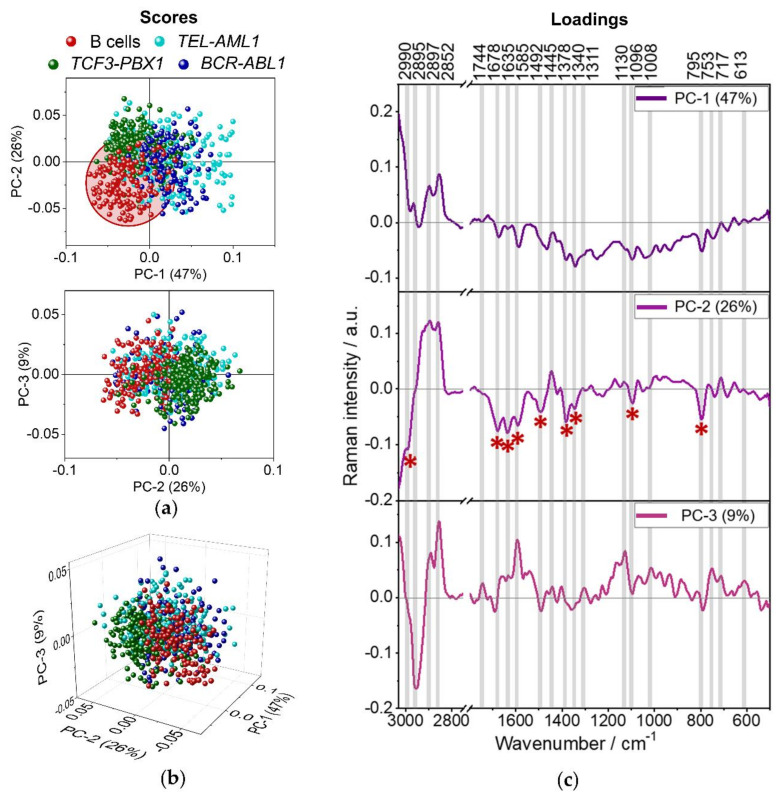
The score plots (**a**,**b**) and loadings (**c**) of PCA analysis representative for B cells and three groups of leukemic cells, in the spectral range of 3030–2750 cm^−1^ and 1800–500 cm^−1^; laser excitation of λ = 532 nm. Color-coded asterisks mark the most pronounced Raman features in the loadings (*).

PCA of spectra representative for the whole B cells (red dots) and three subtypes of leukemic cells collected from patients, i.e., *BCR-ABL1* (blue), *TCF3-PBX1* (green), and *TEL-AML1* (turquoise), is presented in Figure 4.

The left part of Figure 4 presents the score plots, where every data point represents a Raman spectrum of a single cell. The first two score plots are shown along PC-1 to PC-2 and PC-2 to PC-3 axes (Figure 4a). The first three components describe 47%, 26%, and 9% of data variability. In addition, a combined three-dimensional plot for all three PCs, describing in total 82% of data changeability, is presented in Figure 4b. As displayed in Figure 4, B cells show a tendency to group along PC-1 and PC-2 axes. Spectra collected from B lymphocytes are placed mainly on the negative side of both PC-1 and PC-2 axes. In general, the spectra collected from examined leukemic cells mix with each other. Although, a slight trend of grouping can be observed for cells with *TCF3-PBX1* gene fusion along the PC-2 axis. Spectra collected from leukemic cells with *TCF-PBX1* fusion are placed on the negative side of PC-1 and the positive side of PC-2 axes. Loadings of PCA are shown on the right side of Figure 4 and present only those spectral regions where the differences between presented samples are obvious (pointed with color-coded asterisks). Positive/negative Raman features in the loadings indicate increased Raman signals in the original spectra of the positive/negative score values. Raman features present in loadings can indicate the increased intensity of Raman bands in original spectra and changes in the shape and position of Raman bands. Loading for PC-1 does not provide much information about the biochemistry of the cells (Figure 4c). The division along the PC-1 axis is based mostly on variability in a baseline of spectra and probably also on the degree of hydration of the samples. On the PC-2 loading plot, Raman bands characterizing B cells, marked by red asterisks, are negatively correlated with PC-2 (Figure 4c). The most significant are the bands at 1585, 1096, and 795 cm^−1^ (nucleic acids). Other bands worth mentioning are 1492 cm^−1^ (ring breathing of G, A, nucleic acids) and 1378 cm^−1^ (symmetric stretching of COO^−^ groups), 1678 and 1635 cm^−1^ (amide I), indicating the presence of proteins. The band at 2990 cm^−1^ could be associated with lipids, or nucleic acids, whereas 1340 cm^−1^ may indicate the presence of lipids, proteins, or nucleic acids. The high intensity of the nucleic acids and proteins in the mean spectra of the B cells may be related to the morphology of B cells since they contain large nuclei [36,37]. The results also suggest that these cells have more proteins than leukemic cells. Interestingly, the spectra of *TCF3-PBX1* cells tend to group on the negative side of PC-1 and the positive side of PC-2. The bands at 2897 and 2852 cm^−1^ describing symmetric stretching of CH_3_ and CH_2_ groups are characteristic for lipids. The presence of the 1445 cm^−1^ band may indicate the presence of lipids as well (in-plane bending of CH_2_/CH_3_ groups), and 717 cm^−1^ is often distinctive for phospholipids and sphingolipids (vibrations of the choline group). *BCR-ABL1* and *TEL-AML1* cells mostly group on the positive sides of PC-1 and PC-2, which also indicates their more lipidic nature. One can assume that spectra of normal B cells are attributed to more intense bands due to nucleic acids and proteins, whereas leukemic cells show a higher content of lipids.

**Figure 5:**
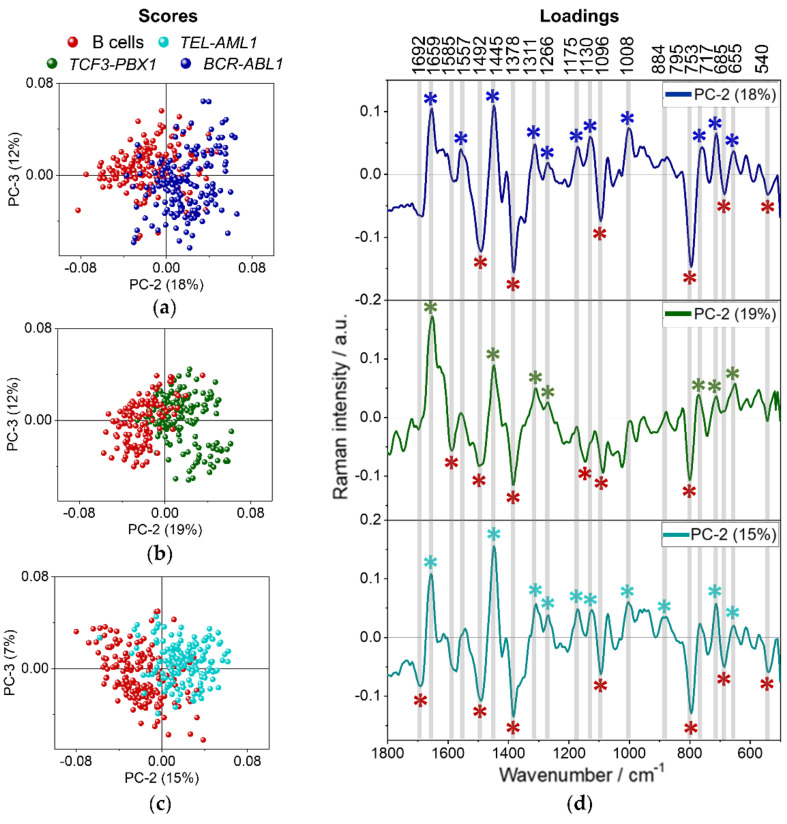
PCA score plots comparing normal B lymphocytes with each leukemia subtype (**a**) *BCR-ABL1*, (**b**) *TCF3-PBX1*, (**c**) *TEL-AML1* and corresponding PCA loadings of PC2 (**d**); laser excitation wavelength of λ = 532 nm. The scores and loadings plots for B cells and *TCF3-PBX1* were multiplied by (−1) for greater clarity of visualization. On each score plot, lymphocytes are represented by red dots and their characteristic bands by red asterisks. Spectra collected from leukemic cells are marked in blue (*BCR-ABL1*), in green (*TCF3-PBX1*), and in turquoise (*TEL-AML1*). Abbreviation a.u.—arbitrary units. The most pronounced Raman features in the loadings are marked by color-coded asterisks (*).

PCA analysis was performed separately for each type of leukemic cell and B lymphocytes to verify whether cancer cells can be distinguished from normal B lymphocytes based on their spectral profiles. The results of PCA are shown in Figure 5. On each score plot, lymphocytes are represented by red dots and their characteristic bands by red asterisks. Analogically, spectra collected from leukemic cells are marked by the following colors: blue (*BCR-ABL1*), green (*TCF3-PBX1*), and turquoise (*TEL-AML1*).

The score plots along PC-2 and PC-3 show significant differences among the four groups of cells. Although PC-1 accounts for the most variance in the data sets, it does not show significant differences across the groups. The score plots present the separation of spectra collected from normal and leukemic cells along PC-2, which describes 15 to 19% of the variability (Figure 5a). Spectra of B cells accumulate on the negative side of PC-2 and are characterized by the negative Raman features in the PC-2 loading plots (Figure 5d). Spectra of leukemia subtypes are agglomerated on the positive side of the PC-2 axis in each score plot and are represented by the bands positively correlated with PC-2 loadings. Each type of neoplastic cell can be well distinguished from B cells. Raman features, which are negatively correlated with PC-2 in the loadings (1585, 1492, 1378, 1096, 795 cm^−1^, Table 1) indicate higher content of nucleic acids in B lymphocytes in comparison to leukemic cells, similarly as presented in Figure 4. PCA revealed several Raman features indicating proteins, including heme: 1311, 1130, 753 cm^−1^ or band at 685 cm^−1^ characteristic for C-C twist vibrations in proteins and guanosine vibrations in nucleic acids as well as the band at 540 cm^−1^ originated from disulfide bonds (Table 1). The malignant cells are described mainly by the bands corresponding to lipids and proteins: 1659, 1445, and 1266 cm^−1^ (Table 1). The band at 717 cm^−1^ indicates the presence of choline (Table 1). Even though such an approach could not discriminate different subtypes of leukemia from each other, slight changes in the loading plots could be noticed. It indicates some chemical variability between the leukemic cells carrying different gene rearrangements. The spectra collected from cells with *BCR-ABL1* gene rearrangement (Figure 5a) additionally can be distinguished from B cells based on the intensity of bands at 1175 and 1557 cm^−1^ (vibrations of Phe, Tyr, and Trp), 1311, 1130, and 753 cm^−1^ (heme proteins: pyrrole asymmetric stretching, C-N stretching, and pyrrole breathing mode, respectively) and 1008 cm^−1^ (ring breathing of phenylalanine, Table 1). Bands associated with heme proteins (1311, 1130, 753 cm^−1^) can also be discriminated from the loading plot describing *TEL-AML1* cells (Figure 5c). The high content of heme proteins in malignant cells was also determined by analyzing the number of cells containing this class of molecules by KMCA (Table 2). It was found in over 60% of leukemic cells. In the case of *TEL-AML1* cells, PCA analysis also revealed a Raman feature at 884 cm^−1^ due to the rocking deformation mode of CH_2_ groups assigned to proteins. When it comes to *TCF3-PBX1*, there are no other distinctive bands (Figure 5b).

In summary, B cells can be distinguished from leukemic cells based on bands assigned to nucleic acids and proteins. In general, malignant cells tend to have more lipidic/protein nature than their normal counterpart. Moreover, cells with *BCR-ABL1* and *TEL-AML1* fusion genes appear to have a higher protein content than *TCF3-PB1*, which are more lipidic. These cells differ the most among all the studied leukemic cell subtypes, which resulted in the most promising grouping of spectra in Figure 5.

### 3.3. Discriminant Analysis of Normal B Lymphocytes and B-ALL Cells

**Figure 6:**
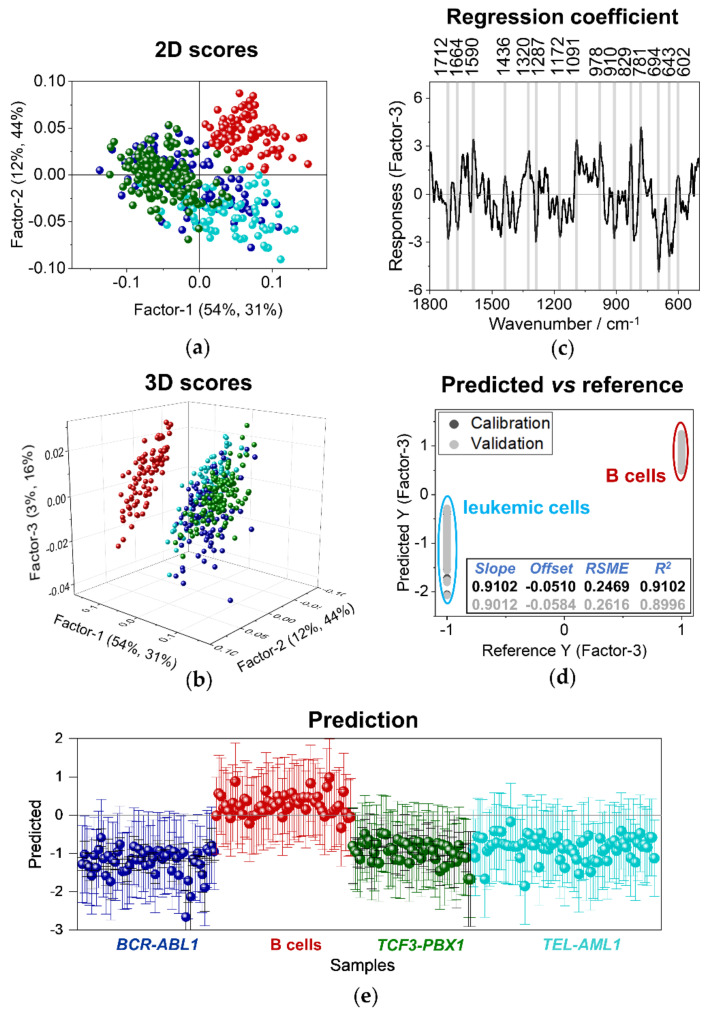
A PLS discriminant analysis. (**a**) Two-dimensional score plot for factors 1 and 2 of PLS-DA. (**b**) Three-dimensional score plot for factors 1–3 of PLS-DA. (**c**) The regression coefficient of the training data set (four groups: B cells, *BCR-ABL1*, *TCF3-PBX1*, and *TEL-AML1*; *n* = 2 for each group). (**d**) Results presenting calibration and validation of the model. (**e**) Prediction results for the test data set (*n* = 1 for each of the following groups: B cells, *BCR-ABL1*, *TCF3-PBX1*, and *TEL-AML1*). Data are obtained for the spectra of whole-cell clusters of B cells and three groups of leukemic cells (*n* = 3 in total for each group); laser excitation of 633 nm.

In order to verify the clinical potential of RS in the diagnosis of leukemia, we employed partial least squares (PLS) discriminant analysis. The goal was to build a model that enables distinguishing and categorizing the spectra obtained from biological samples of healthy donors and patients with ALL disease. Figure 6 presents the results of the PLS model for discriminating spectra of healthy B cells and malignant ones obtained using laser excitation of 633 nm.

**Figure 7:**
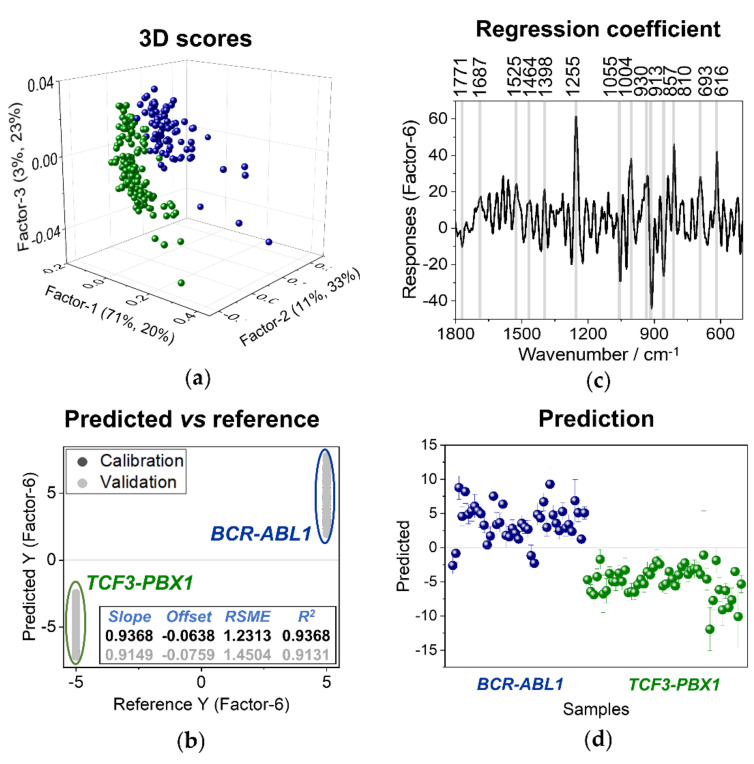
A PLS discriminant analysis. (**a**) Two-dimensional score plot for factors 1 and 2 of PLS-DA. (**b**) Results presenting calibration and validation of the model. (**c**) The regression coefficient of the training data set (two groups: *BCR-ABL1* and *TCF3-PBX1*; *n* = 3 for each group). (**d**) Prediction results for the test data set (*n* = 3 for each of the following groups: *BCR-ABL1* and *TCF3-PBX1*). Data are obtained for the spectra of whole-cell clusters of two groups of leukemic cells (*n* = 3 in total for each group); laser excitation of 633 nm.

First, a PLS model in the spectral range of 1800–500 cm^−1^ has been established comparing leukemic cells (*BCR-ABL1*, *TCF3-PBX1*, and *TEL-AML1*) with normal lymphocytes (Figure 6) and cancers cells with *BCR-ABL1* and *TCF-PBX1* mutations (Figure 7). With respect to the data presented in Figure 6, the training set included spectra of B lymphocytes and neoplastic cells (*BCR-ABL1*, *TCF3-PBX1*, and *TEL-AML1* gene mutations) collected from samples derived from two out of three patients. The training set presented in Figure 7 included 75% of spectra of leukemic cells collected from samples obtained from three patients to avoid the influence of individual variability and other factors that could diminish classification. By building the model presented in Figure 6, spectra of the malignant cells and B lymphocytes were assigned the value of (−1) and (1), respectively. For a model aimed at differentiating between leukemic cells, spectra of *TCF-PBX1* and *BCR-ABL1* cells, the value of (−5) and (5) were assigned, respectively (Figure 7).

The results of the 3-factor PLS model for distinguishing spectra of the whole-cell body of healthy B cells and malignant ones obtained using laser excitation of 633 nm are presented in Figure 6. Results presented in PLS two-dimensional calibration plot, including factors 1 and 2 (Figure 6a), confirmed that there are, in fact, distinct differences between B cells (red dots) and cells derived from patients diagnosed with B-ALL. Again, a tendency of B cells to group together can be noticed. The three-dimensional calibration score plot of factors 1–3 (Figure 6b) delivers more information about the model. It appears that there is a slight tendency of the grouping of spectra of leukemic cells, noticeable especially for the cells with *TCF3-PBX1* fusion gene (green). There is also a similar trend of grouping spectra of cells with *BCR-ABL1* (blue) and *TEL-AML1* (turquoise) gene rearrangements. However, cells with *BCR-ABL1* (blue) and *TEL-AML1* (turquoise) fusions have more heterogeneous characteristics. In Figure 6c, a plot of the regression coefficient is presented. It shows which bands discriminate two analyzed groups of cell spectra, Raman spectra of normal B cells and their malignant counterparts. Bands appearing on the positive side of this plot characterize B cells, whereas leukemic cells are on the negative side of the plot. Normal lymphocytes exhibit Raman bands at 1590, 1320, 1091, 827, and 781 cm^−1^, corresponding to nucleic acids, and the band at 1436 cm^−1^, which can be assigned to both lipids and proteins. The bands distinguishing leukemic cells from the normal ones are observed at 1664 (amide I, proteins), 1172 (Tyr, Phe, proteins), and 694 cm^−1^ (C-C twist, proteins). Albeit the regression coefficient is relatively low, obtained PLS results agree with the one from the PCA analysis. However, the PLS method’s advantage is that it provides a tool for categorizing Raman spectra of cells and predicting whether a spectrum has been recorded for a normal or cancer cell. Additionally, an established PLS model can be used to categorize spectra of unknown samples. Figure 6d shows predicted vs. reference values of responses for the training data set. For the model, values of R^2^ are equal to 0.9102 for calibration and 0.8996 for validation. As the next step, spectra of one sample of each studied group were incorporated in the evaluation of the model. The prediction plot (Figure 6e) indicates that the obtained model can distinguish healthy B cells from neoplastic ones. Spectra of malignant cells were positioned on the negative side of the chart, approximately around the value of (−1), assigned to leukemic cells during the algorithm’s training. The prediction coefficient R^2^ was equal to 0.75. The model achieved the sensitivity of 100% (calculated as true positives divided by the sum of all positives) and specificity of 83% (computed as true negatives in relation to all negatives).

Since each of the analyzed subtypes of leukemia could be distinguished from the normal B cells (Figure 4, Figure 5 and Figure 6), we investigated if PLS enables us to notice any differences in Raman spectra between each leukemia subtype. For this purpose, we performed PLS analysis comparing malignant cells carrying different gene abnormalities. The most promising model differentiating cells with *BCR-ABL1* and *TCF-PBX1* rearrangements is presented in Figure 7. The bands distinguishing leukemic cells with *BCR ABL1* mutation from cells with *TCF-PBX1* gene alteration can be mainly attributed to bands that originated from proteins at 1687 (amide I, proteins), 1255 (amide III, proteins), 1004 (ring breathing of phenylalanine), 930 (N–Cα–C vibration, proteins), 693 (C-C twist, proteins), and 616 cm^−1^ (ring breathing of phenylalanine). On the other hand, bands characterizing cells with *TCF-PBX1* mutation could be assigned to different bands that originated from the proteins modes observed at 1275, 1255 (amide III, proteins), 1055 (C-C vibrations of phenylalanine), 913 (C-C vibrations of phenylalanine), 857 cm^−1^ (tyrosine). The differences in Raman fingerprints of cells carrying different genetic mutations related to protein-specific content (cytochrome, Phe, Tyr, Trp) suggest diversities in the metabolism of blasts carrying different genetic aberrations. The tyrosine Raman feature at 857 cm^−1^, which characterizes *TCF-PBX1* cells, can be related to altered tyrosine phosphorylation in cells with the *Philadelphia* chromosome because due to tyrosine phosphorylation, Fermi resonance doublet collapses to a single band at 830 cm^−1^ [38]. The presence of the band characteristic mainly for RNA at 810 cm^−1^ in *BCR-ABL1* may also indicate different transcriptional and translational activity of *Ph-positive* cells.

## 4. Discussion

Rapid and label-free identification of malignant cells from patients suffering from leukemia is an unwavering need within health care prospects. Therefore, the primary goal of our study was to identify whether Raman imaging conjugated with multivariate analysis (PCA or PLS) can be used as a distinguishable tool between normal cells *vs*. their malignant counterparts and, more importantly, in layout concerning discrimination of diverse subtypes of BCP-ALL.

Firstly, our results clearly indicate spectra separation of B cells from their malignant counterparts. The PCA scores plots confirmed discrimination between BCP-ALL cells and healthy B lymphocytes, thereby confirming the possibility of developing a classification model to distinguish these groups of cells. Such an approach was previously demonstrated only for experimental outlines based on leukemic cell lines [39]. The PLS model established within the study allowed cell spectra of *BCR-ABL1*, *TCF3-PBX1,* and *TEL-AML1* to be correctly classified as leukemic cells with 100% accuracy. Analogous analysis in a group of healthy B cells reached the maximum specificity equal to 83%, with a 17% margin of B cells incorrectly classified as malignant. The model developed within our study admittedly allowed the diagnosing of neoplastic cells but indisputably needs improvement to categorize normal cells correctly and increase the maximal level of specificity. For this purpose, the algorithm should be trained on a significantly bigger data set to eliminate individual variability.

The bands characterizing normal B cells and which determined discrimination included: 1492, 1378, 1096, 795, 685 cm^−1^ (Figure 4 and Figure 5). They can be assigned to nucleic acids. The decrease of these bands in leukemic cells indicates a decrease of the nucleic-acid content in lymphoblasts. Our findings are in line with the previous results [18,19,20,40]. Managò et al. indicated that Raman bands associated with vibrational modes characteristic for nucleic acids could be successfully used as spectroscopic markers to distinguish normal and cancer cells. The accuracy of the discrimination based on the PCA analysis was at the level of 96%. Moreover, the 1447 cm^−1^/785 cm^−1^ ratio was higher for clinical samples and leukemic cell lines than B lymphocytes [20]. Our results show the same tendency (Figure S2). It was previously pointed that the reduction in the nucleic-acid content in leukemic cells might be observed due to chromatin decondensation and higher levels of transcriptional and replicational activities in cancers cells and/or breaks and translocations of chromosomes [20,40]. The change in the degree of chromatin condensation may also be indicated by changes in the morphology of the neoplastic cell nucleus compared to normal B cells (*TCF3-PBX1* and *TEL-AML*), as reflected by the calculated ratio of the nucleus area to the whole cell area (Figure S3). Managò et al. also showed that individual variability strongly impacts spectral discrimination between normal and cancer cells, which was also observed in our study [20].

Secondly, we attempt to distinguish cells representing different subtypes of BCP-ALL derived from patients (*BCR-ABL1*, *TCF3-PBX1*, and *TEL-AML*). However, we could not obtain the precise key allowing for the distinction between subtypes of BCP-ALL (Figure 4). The loadings plot in Figure 5 presents very similar shapes of bands and loadings for malignant cells, which currently rule out the possibility of obtaining division. Nevertheless, in the Raman spectra, some subtle changes can be seen. The loading plots for all aberrations present the bands at 1659, 1445, 1311, 1266, 753, 717, and 655 cm^−1^, which can be assigned to lipids and proteins. Similar conclusions were drawn from studies reported in the literature [20]. Due to higher levels of transcriptional activity in metabolically active cancer cells, an increase in proteins level may be correlated with increased biomolecular synthesis. Yet, there are several bands characteristic only for specific subtypes. In the case of *BCR-ABL1* and *TEL-AML1* fusion genes, there are significant bands, which can be assigned to hemoproteins, i.e., 1311, 1130, and 753 cm^−1^. Nonetheless, these two mutations could potentially be distinguishable since BCR-ABL1 was also characterized by the 1557 and 1008 cm^−1^ bands, whereas the latter subtype provides a band at 884 cm^−1^. These three bands are assigned to proteins, but they are present on loading plots of different mutations. This may indicate differences in protein composition in cells with these fusion genes [41,42]. Regarding the cells isolated from patients with *TCF3-PBX1* gene rearrangement, there are no additional marker bands. Even though we could not establish a model discriminating all studied leukemic subtypes of leukemia, we achieved a promising model differentiating cells with *BCR-ABL1* and *TCF-PBX1* gene alterations (Figure 7). There are several reasons, including individual variability, why the use of spontaneous Raman spectroscopy makes it difficult or even impossible to distinguish between genetic subtypes of B cell precursor leukemia at a similar stage of differentiation. According to the FAB classification, cells with the same gene rearrangement may exhibit different morphology defined as L1 or L2 subtypes [43]. Moreover, within the specific subtype of BCP-ALL leukemia, additional coexisting genetic lesions may influence the analyzed cells’ phenotypic diversity and make classification complicated. Finally, biochemical changes in leukemic cells of different subtypes may be so subtle that they are very difficult to be detected by spontaneous Raman spectroscopy.

Our analysis showed differences in lipid content within BCP-ALL subtypes and described *TCF3-PBX1* with the highest lipid content. The altered lipidic profile was reported previously between normal and leukemic cells, where the higher abundance of lipids in cancer cells was correlated with the abnormal growth regulation of leukemic cells [44]. The uptake of free acid acids in the form of lipid droplets or their incorporation into triglycerides and phospholipids by ALL cells was previously disclosed [45]. The presence of lipid droplets in the larger amount in leukemic cells compared to B-cells and the highest amount in *TEL-AML*-positive cells might indicate the correlation of the number of stored lipids with the metabolic activity of the leukemic cells.

Another part of the results obtained within this work showed the presence of hemoproteins in cells of all four ALL subtypes, but in different proportions. The lowest number of cells with hemoproteins content was observed within B lymphocytes (56.5%), and successively, such number increased in BCP-ALL lymphoblast carrying *TEL-AML1* (60.2%), *TCF3-PBX1* (63.4%), and *BCR-ABL1* (67.8%). Those differences align with phenomena in which leukemic cells tend to have a higher level of proteins containing hem as a prosthetic group (e.g., ferroporphyrins) since they are described as rapidly proliferating with altered metabolism. However, the viability of *BCR-ABL1* cells was the lowest among all studied subtypes of B-ALL (43–56%, Table S1). Nevertheless, the highest percentage of hemoproteins in *BCR-ABL1* cells did not result from apoptosis because apoptosis might be marked by the decrease of Raman signals assigned to cytochrome c [46,47]. In the case of lymphoma, it was shown that an increased expression of cytochrome c was associated with poor patient prognosis, and it was significantly correlated with an outcome predictor score, accurately anticipating patient survival following chemotherapy [48]. Considering poor prognosis in the case of *Philadelphia*-positive leukemias, an increased level of hemoproteins in the *BCR-ABL1* subtype of ALL could reflect its invasiveness. It may be rather associated with a stronger proliferative potential of *BCR-ABL1* cells than *TCF-PBX1* or *TEL-AML1* fusions. In the future, assessing the level of hemoproteins in leukemic cells might be a promising diagnostic factor for B-ALL subtypes.

The 633 nm laser excitation measurements allowed us to detect carotenoids in all fractions of cells representing BCP-ALL subtypes. The highest content of carotenoids of 12.5% was found in *TEL-AML1,* with a downward trend observed in *TCF3-PBX1* (11.8%) and *BCR-ABL1* (8.35%). Carotenoids cannot be synthesized in the human organism and thus must be provided with food. They accumulate in particular cells, have immunomodulatory activity, and can be transformed into, e.g., retinol [49]. Previous studies proved that those compounds could be found in human T cells but not in B lymphocytes [33,35,49,50,51], which aligns with our previous research. These findings might suggest that leukemic cells, which originate from pre-B cells, abnormally accumulate carotenoids. Their changed metabolism might cause this and could possibly become a marker of leukemia development.

The use of two lasers of different excitations allowed us to collect full spectral information and perform a complete biochemical analysis of both normal B cells and lymphoblasts. Spectra obtained with a 532 nm laser excitation show a better signal-to-noise ratio for heme proteins due to resonance enhancement. Applied excitation allows the application of a smaller sampling density, so visualizing lipid droplets is possible. However, under these conditions, strong absorption accompanies light scattering, which is decreased when the measurement is performed with a 633 nm laser [52]. The 633 nm laser has proven to be more powerful in studies of carotenoid accumulation in leukemic cells due to photosensitivity of this group of molecules [53] and a resonance Raman enhancement phenomenon, which occurs when excited by radiation from the range of 400–550 nm [52,53].

Summarizing, our results indicate wide spectra of ambiguities accompanied by the application of Raman spectroscopy in the advanced biology of blood malignancies. The question posed in our study whether spontaneous Raman spectroscopy is an efficient tool to distinguish genetic subtypes of B cell precursor leukemia turned out to be a challenging goal to reach. However, it revealed a wider perspective of the subject where the Raman phenomenon is important and the biological context of analyzed cells. Moreover, one of the facts worth emphasizing is that all cells used herein were at the same stage of differentiation (pre-B common and pre-B-ALL), thereby posing the same shape or analogous ratio of nucleus to the cytoplasm (Figure S3). Therefore, the golden key to successful differentiation probably lies in subtle features within the biochemical frame but probably does not change cancer cell morphology. This suggests that one should look for alternative chemometric methods for data analysis or consider introducing Raman reporters or markers reflecting molecular/biochemical differences/features that may exaggerate the differences between different leukemia subtypes/differentiate the cellular metabolism features of different leukemia subtypes [54]. So far, such reporters are used in targeting specific cellular structures, such as MitoBADY, which accumulates in the mitochondria, or EdU, which bonds with DNA [54]. Characteristics and proper segregation of common BCP-ALL into subtypes probably require a wide spectrum of immunophenotyping methods, cytogenetics, and molecular diagnostics. Gene expression profiling using microarrays identify differentially expressed genes that correlate with lineage and primary genetic change. Anderson et al. showed that most of the analyzed ALL lymphoblasts samples segregate according to the primary genetic aberrations group [55]. Identifying upregulated genes, which correlates with primary genetic changes, gives information of key processes with variable efficiency in different genetic leukemia subtypes [55].

While we have achieved reproducible measurements that allowed us to distinguish between normal B cells and B-ALLs, several limitations remain. We anticipate that given the low degree of biochemical variation between some leukemia subtypes in terms of general protein and lipid composition, it would be difficult to discriminate them by PCA or PLS models. Therefore, to identify reliable and robust spectral differences between ALL subtypes, a more advanced prediction model based on deep learning and a supervised approach is needed. There are also other limitations in the translation of Raman-based diagnostic of leukemia from the laboratory into the clinic, such as mentioned above difficulties in analyzing the data and relatively slow acquisition times. The development of non-linear Raman methods provides improvements in collection times for conventional Raman spectroscopy and has been shown to significantly reduce acquisition times: coherent anti-Stokes Raman spectroscopy (CARS) and stimulated Raman spectroscopy (SRS) [56].

The data collected by different research approaches might be used to design the supervised data analysis based on Raman spectroscopy imaging which focuses on previously described alterations at the genetic and proteomic levels of signaling pathways analysis.

## 5. Conclusions

B cells from healthy donors and three genetic subtypes of BCP-ALL were analyzed using Raman spectroscopy. PCA indicated that B cells significantly differ from malignant cells. This led us to a successfully trained PLS algorithm to recognize healthy B lymphocytes from leukemic cells. We achieved a sensitivity of the model equal to 1.00 and specificity of 0.83, which means that the algorithm can be used to recognize true positive cells. Still, about one-sixth of healthy cells are classified as malignant. Such results prove that Raman imaging supported with chemometric analysis can be considered an innovative tool in leukemia diagnostics.

## Figures and Tables

**Table 1:** Assignments of the Raman bands in the spectra of studied samples.

BAND/cm^−1^	Assignment	Classification
540	Disulfide bonds [23]	Proteins
613	Ring breathing of Phe [24,25,26]	Proteins
655	Ring breathing of Phe and Tyr [25,26]	Proteins
685–694	C-C twist, G [19]	Proteins, nucleic acids
717	Vibrations of the choline group [27]	Phospholipids, sphingolipids
728	Ring breathing of A [4]	Nucleic acids
753	Pyrrole breathing mode [28,29]	Heme proteins
795	Ring breathing of U, T, C; backbone O-P-O [4]	Nucleic acids
827	PO_2_^−^ asymmetric stretching, Tyr [19,29,30]	Nucleic acids, proteins
884	CH_2_ rocking deformation mode [4]	Proteins
940	Skeletal modes [4] N–Cα–C for α-helix [31]	Polisaccharides Proteins
1008	Ring breathing of Phe [4]	Proteins
1039	C-N stretching [4]	Proteins
1096	PO_2_^−^ symmetric stretching [4,19]	Nucleic acids, phospholipids
1130	C–C, C–O, C-N stretching [31,32]	Heme proteins, proteins, lipids
1162	C-C stretching [33]	Carotenoids
1175	Tyr, Phe, C-H bend	Proteins
1266	=C−H deformation, amide III; C-N stretching in α-helix [4]	Lipids, proteins
1311	CH_2_ twist, C-N stretching, Cα-Cβ stretching [19,32]	Lipids, proteins, heme proteins
1340	CH_2_/CH_3_ wagging, twisting and/or bending; CH deformation, G modes [4,19]	Lipids Proteins Nucleic acids
1378	COO^–^ symmetric stretching [30,34]	Proteins, nucleic acids
1449–1455	CH_2_/CH_3_ bending in-plane [4]	Lipids, proteins
1492	Ring breathing of G, A, CH deformation mode [19]	Nucleic acids Nucleic acids, proteins
1525	C=C stretching [33]	Carotenoids
1557	Phe and Trp [30]	Proteins
1585	Pyrimidine ring; C=C bending in Phe [4]	Nucleic acids, heme proteins
1635–1670	Amide I, C=C stretching [4,19,32]	Proteins, lipids
2850–2870	CH_2_ symmetric stretching [4]	Lipids, fatty acids
2885	CH_3_ symmetric stretching, CH_2_ asymmetric stretching [4]	Lipids, fatty acids, proteins
2933–2945	CH_3_ asymmetric stretching [4]	Lipids, fatty acids, proteins
3010	=CH_2_ stretching [4]	Lipids, fatty acids

**Table 2:** A summary of measurements using two lasers (λ = 532 nm and λ = 633 nm), including the number of donors (N), number of measured cells, and percentage of cells with heme proteins, carotenoids, and lipid clusters recognized by KMC. The values representing the most abundant cluster within the analyzed cells are in bold.

CELLS	N	λ [nm]	Number of Cells	Heme Proteins Clusters	Carotenoid Clusters	Lipid Clusters
B cells	5	532	255	144 (56.5%)	0 (0.0%)	3 (1.2%)
		633	247	0 (0.0%)	0 (0.0%)	0 (0.0%)
*BCR-ABL1*	3	532	183	**124 (67.8%)**	0 (0.0%)	5 (2.7%)
		633	169	0 (0.0%)	14 (8.3%)	7 (4.1%)
*TCF3-PBX1*	4	532	331	210 (63.4%)	8 (2.4%)	9 (2.7%)
		633	271	4 (1.5%)	32 (11.8%)	2 (0.7%)
*TEL-AML1*	4	532	314	189 (60.2%)	**16 (5.1%)**	**66 (21.0%)**
		633	257	3 (1.2%)	**32 (12.5%)**	**2 (1.1%)**

## Data Availability

Derived data supporting the findings of this study are available from the corresponding author on reasonable request.

## Supplementary Materials

The following are available online at https://www.mdpi.com/article/10.3390/cancers13215483/s1, Figure S1: Purity assessment of the B cell fraction by flow cytometry, Figure S2: The 1445 cm^−1^/795 cm^−1^ ratio calculated based on the spectra obtained with the use of 532 nm laser for B cells and lymphoblasts, Figure S3: The nucleus/cell ratio calculated based on the KMC maps obtained with the use of 532 nm laser for B cells and lymphoblasts, Table S1: Details of samples used in the study.
