# Supplementary material for: Towards Raman-Based Screening of Acute Lymphoblastic Leukemia-Type B (B-ALL) Subtypes
Source: Cancers (Basel). 2021 Oct 31;13(21):5483. doi: 10.3390/cancers13215483 (PMC8582787; doi:10.3390/cancers13215483)
Supplement: Supplementary file 1 [file cancers-13-05483-s001.zip › cancers-1413071-SI.pdf]

cancers

MDPI

Supplementary Information

Supplementary Table S1. Details of samples used in the study.

Sample ID	Immunophenotype	Type of gene rearrangements	Blastosis on diagnosis	Cell viability
P1	pre-B common	TEL-AML1	65%	70%
P2	pre-B common	TEL-AML1	98%	81%
P3	pre-B	TEL-AML1	91%	81%
P4	pre-B common	TEL-AML1	93%	73%
P5	pre-B common	BCR-ABL1	83%	56%
P6	ND	BCR-ABL1	ND	43%
P7	pre-B common	BCR-ABL1	91%	55%
P8	pre-B	TCF3-PBX1	95%	88%
P9	pre-B	TCF3-PBX1	86%	50%
P10	pre-B common	TCF3-PBX1	94%	84%
HV1	normal lymphocyte B	-	-	96%
HV2	normal lymphocyte B	-	-	86%
HV3	normal lymphocyte B	-	-	78%
HV4	normal lymphocyte B	-	-	98%
HV5	normal lymphocyte B	-	-	96%

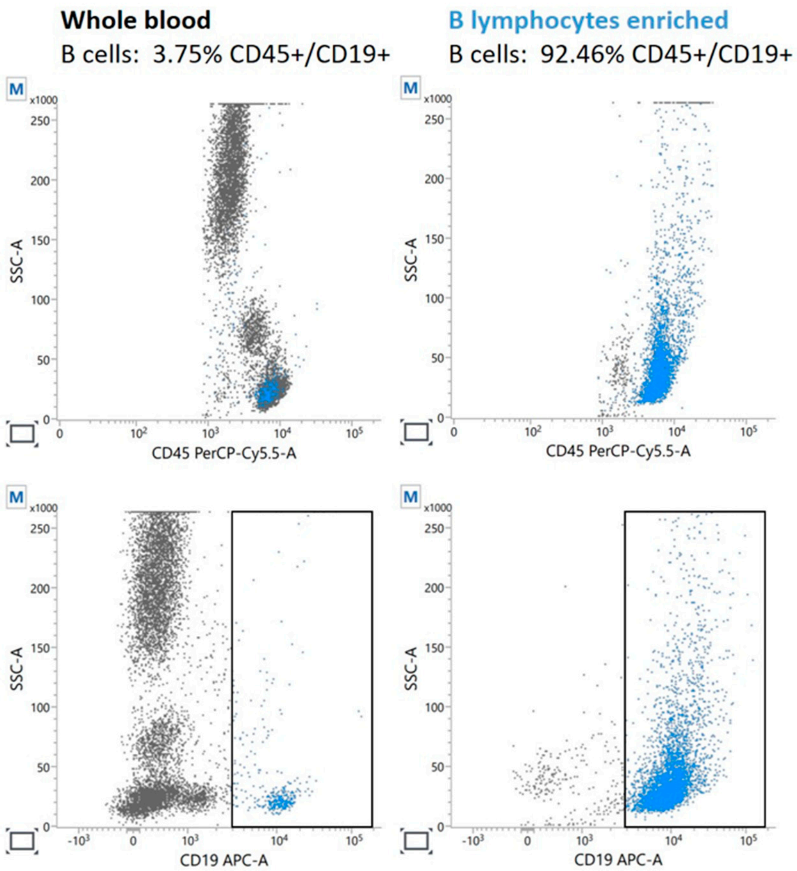

Supplementary Figure S1. Purity assessment of the B cell fraction by flow cytometry. Before and directly after the magnetic sorting procedure, B cell fraction was subjected to purity assessment. The lymphocyte population was identified based on side-scatter (SSC) characteristics and CD45 expression. In the whole blood, B cells were defined further as CD19-expressing cells in the CD45-positive population. The initial concentration of CD19+ cells in the blood of one healthy volunteer was 3.75%. After magnetic separation, the purity of enriched lymphocytes was assessed within CD45 positive leucocytes and exceeded 90%. The picture presents the analysis of one sample.

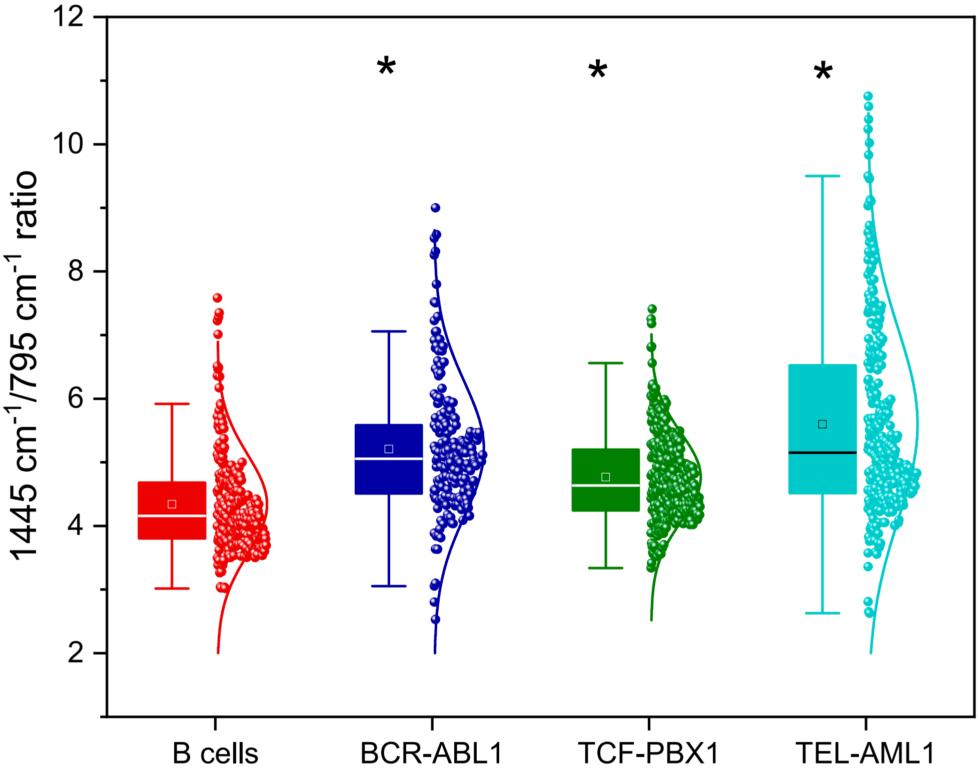

Supplementary Figure S2. The 1445 cm^{-1}/795 cm^{-1} ratio calculated based on the spectra obtained with the use of 532 nm laser for B cells (n=250), and *BCR-ABL1* (n=180), *TCF3-PBX1* (n=326), *TEL-AML1* (n=302) lymphoblasts. Due to the lack of a normal distribution, a non-parametric was used to test the significance of changes with respect to normal cells (Mann-Whitney U test, $p < 0.05$).

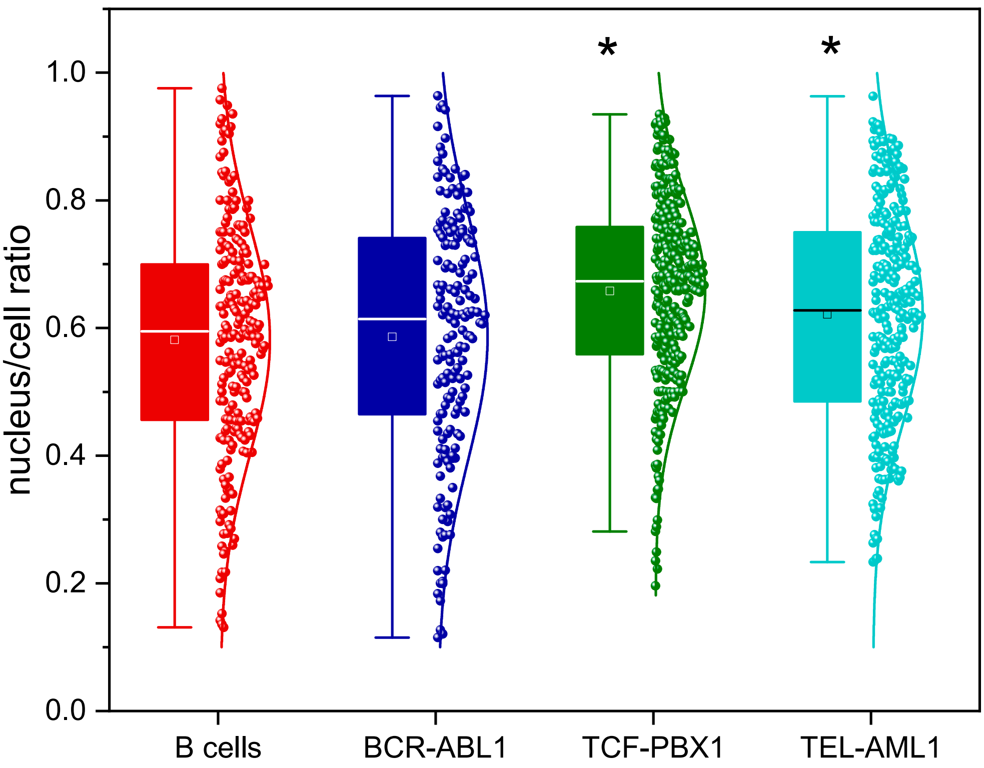

Supplementary Figure S3. The nucleus/cell ratio calculated based on the KMC maps obtained with the use of 532 nm laser for B cells (n=254), and *BCR-ABL1* (n=183), *TCF3-PBX1* (n=331), *TEL-AML1* (n=314) lymphoblasts.

Due to the lack of a normal distribution, a non-parametric was used to test the significance of changes with respect to normal cells (Mann-Whitney U test, $p < 0.05$).
